# Antidepressant-Like Activity of 10-Hydroxy-Trans-2-Decenoic Acid, a Unique Unsaturated Fatty Acid of Royal Jelly, in Stress-Inducible Depression-Like Mouse Model

**DOI:** 10.1155/2012/139140

**Published:** 2011-07-24

**Authors:** Satoru Ito, Yuji Nitta, Hidefumi Fukumitsu, Hitomi Soumiya, Kumiko Ikeno, Tadashi Nakamura, Shoei Furukawa

**Affiliations:** ^1^Laboratory of Molecular Biology, Department of Biofunctional Analysis, Gifu Pharmaceutical University, Daigaku-nishi Gifu 501-1196, Japan; ^2^Japan Beekeeping Co. Ltd., Kanofujimachi, Gifu 500-8471, Japan

## Abstract

Symptoms of depression and anxiety appeared in mice after they had been subjected to a combination of forced swimming for 15 min followed by being kept in cages that were sequentially subjected to leaning, drenching, and rotation within 1-2 days for a total of 3 weeks. The animals were then evaluated by the tail-suspension test, elevated plus-maze test, and open-field test at 1 day after the end of stress exposure. Using these experimental systems, we found that 10-hydroxy-trans-2-decenoic acid (HDEA), an unsaturated fatty acid unique to royal jelly (RJ), protected against the depression and anxiety when intraperitoneally administered once a day for 3 weeks simultaneously with the stress loading. Intraperitoneally administered RJ, a rich source of HDEA, was also protective against the depression, but RJ given by the oral route was less effective. Our present results demonstrate that HDEA and RJ, a natural source of it, were effective in ameliorating the stress-inducible symptoms of depression and anxiety.

## 1. Introduction

Royal jelly (RJ), which is fed to the queen honeybee, has a variety of biological activities towards various types of cells. For instance, RJ exhibits immunomodulatory properties [1–3] and inhibits the development of atopic dermatitis-like skin lesions [4]. Earlier we found that RJ has the ability to induce neurites from cultured rat pheochromocytoma PC12 cells and identified an adenosine monophosphate (AMP) analog, AMP N^1^-oxide, as an active entity [5]. As AMP N^1^-oxide is a unique compound of RJ, the effects of RJ on the nervous system may be attributed to this compound. Further, RJ contains another unique component, 10-hydroxy-trans-2-decenoic acid (HDEA), an unsaturated fatty acid [6]. HDEA has been reported to have many pharmacological activities such as antitumor activity [7], collagen production-promoting activity [8], and antibiotic activity [9]. We recently found that HDEA increases neurogenesis, but decreases glial generation, of cultured neural stem/progenitor cells (NSCs) [10] as well as the production of neurotrophins including brain-derived neurotrophic factor (BDNF) [11]. These results prompted us to test the therapeutic effect of HDEA on depression, because significant neurogenesis in the hippocampus suggests a mechanism through which BDNF might be related to depression [12]. 

 The hippocampi of patients who suffer from depression are significantly smaller than those of healthy individuals [13]. This may be due to the decreased neurogenesis in depressed individuals, because hippocampal neurogenesis is reduced by stress [12] and increased by antidepressant treatment [14]. The parallel changes in BDNF levels and neurogenesis in response to stress and antidepressant treatment suggest a positive correlation between the BDNF level and hippocampal neurogenesis. These lines of evidence have led to the neurotrophic hypothesis of depression [15], suggesting that HDEA and RJ, a source of it, might influence ultimately vulnerability to depression. HDEA and RJ are safe natural products, and the latter is widely known and popular as a health food throughout the world. Thus they might be useful to protect against depression. 

 Therefore, we tested the protective effect of HDEA and RJ on stress-induced depression or anxiety of model mice and, as expected, found them to be efficacious.

## 2. Materials and Methods

### 2.1. Animals

Seven-week-old male ddY mice (Japan SLC, Hamamatsu, Japan), weighing 35–40 g, were used. The mice were housed under conditions of constant temperature (23 + 2°C), humidity (55 + 10%), and a 12 h light/12 h dark cycle with food and water available freely. All animal experiments were performed according to the Guideline for Care and Use of Laboratory Animals of Gifu Pharmaceutical University. 

### 2.2. Drug Treatment

**Figure 1:**
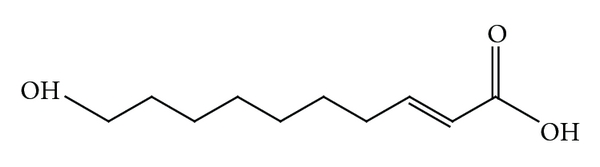
Chemical structure of HDEA.

RJ (originated from *Apis melifera*) and 10-hydroxy 2-decenoic acid (HDEA; Figure 1) were provided by Japan Beekeeping Co. Ltd. (Gifu, Japan). For comparison with a popularly prescribed antidepressant, we also used fluvoxamine, purchased from Sigma Chemicals. During stress exposure, RJ (250 mg/kg/day), HDEA (100 or 500 *μ*g/kg/day), or fluvoxamine (1000 *μ*g/kg/day), each dissolved in phosphate-buffered saline (PBS), was injected intraperitoneally into mice once a day for 21 days from the onset of the stress loading. Alternatively, RJ (2.5 g/kg/day) was orally administered to the animals via a metal gastric zonde.

### 2.3. Stress-Induced Depression-Like Model Mice

Stress-induced depression-like model mice [16] were prepared by a combination of modified forced swimming [17, 18] and chronic mild stress (CMS) [19]. Briefly, the mice were individually placed into 5-L glass beakers (height 27 cm, diameter 18 cm) filled with 4 L of water (23 + 1°C) and kept there for 15 min. After that, the mice were removed and dried with a drier before being returned to their home cages. Two days later, the caged mice were exposed to CMS, which consisted of 3 different and sequential stress situations: inclining their cage by 20 degrees from the horizontal (CMS 1), keeping them on chip bedding wetted with 200 ml of water (CMS 2), and shaking the cages at 180 rpm by a rotatory shaker (CMS 3). These stress situations were applied for 48, 24, and 24 hr, respectively, with a 24 hr interval between each situation.

### 2.4. Elevated Plus-Maze Test

This is a standard test of fear and anxiety. After treatment, the animals were placed in the center of a 4-arm maze (30 × 5 cm/arm) elevated to a height of 50 cm, in which 2 arms were open and 2 were enclosed [20]. The number of times the animal entered each of the arms and the time spent in each arm were recorded during a 5-min test period. The procedure was conducted in a sound-attenuated room.

### 2.5. Open-Field Test

The open-field test is another test for fear and anxiety, utilizing a square arena 96 × 96 cm^2^ with 60 cm high walls. The floor was divided into 16 squares by parallel and intersecting white lines [21]. Four squares were defined as the center and the 12 squares along the walls, as the periphery. Mice were placed in the center of the field and allowed to explore freely for 5 min. The percent of time spent in the center areas of the field was calculated, and number of squares crossed was counted. 

### 2.6. Tail-Suspension Test

The tail-suspension test is a standard assay for depression because decreased motivation is a hallmark symptom [22]. In this test, a mouse was suspended by its tail from a hanger attached to a precision linear load cell. Although measurements were taken for 7 min, immobility was calculated by determining the time spent immobile during the last 6 min of the test, because all mice were uniformly active for the first min. Immobility time was scored by a blinded observer. Mice that climbed their tail or fell off the hanger were excluded from analysis.

## 3. Results

### 3.1. Effect of HDEA on the Stress-Induced Depression

**Figure 2:**
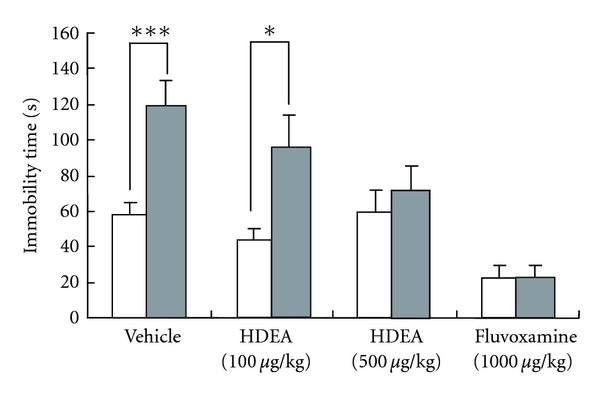
Effects of HDEA on depression in mice. The mice were daily injected intraperitoneally with vehicle, HDEA (100 or 500 *μ*g/kg) or fluvoxamine (1 mg/kg) for 3 weeks with simultaneous exposure to the stress described in Materials and Methods and then subjected to the tail suspension test at 24 hr after the end of the stress. Significance of differences from the values of mice without stress exposure (control) was determined by performing Student's *t*-test (*P < 0.05, ***P < 0.001).

Prior to stress loading, mice were evaluated for immobility time during the last 6 min of the tail-suspension test; and those with suspiciously low or high values (i.e., outliers) were excluded from the study in order to minimize the variability of immobility time. The remaining mice (*n* = 8/group) were administered vehicle, HDEA (100 *μ*g/kg), HDEA (500 *μ*g/kg), or fluvoxamine (1000 *μ*g/kg) once a day for 21 days during the stress loading; then the tail-suspension test was performed (Figure 2). The immobility time of the stress-loaded animals was 2 times greater than that of the nonstress-loaded animals in the vehicle-treated group, indicating that the stress caused depression. A similar result was obtained for the low-dose HDEA-treated group. However, this significant difference in immobility time between stress loading and nonstress loading disappeared in the high-dose HDEA-treated and fluvoxamine-treated groups, suggesting that the high-dose HDEA and fluvoxamine effectively protected against depression.

### 3.2. Effect of HDEA on the Stress-Induced Anxiety

**Figure 3:**
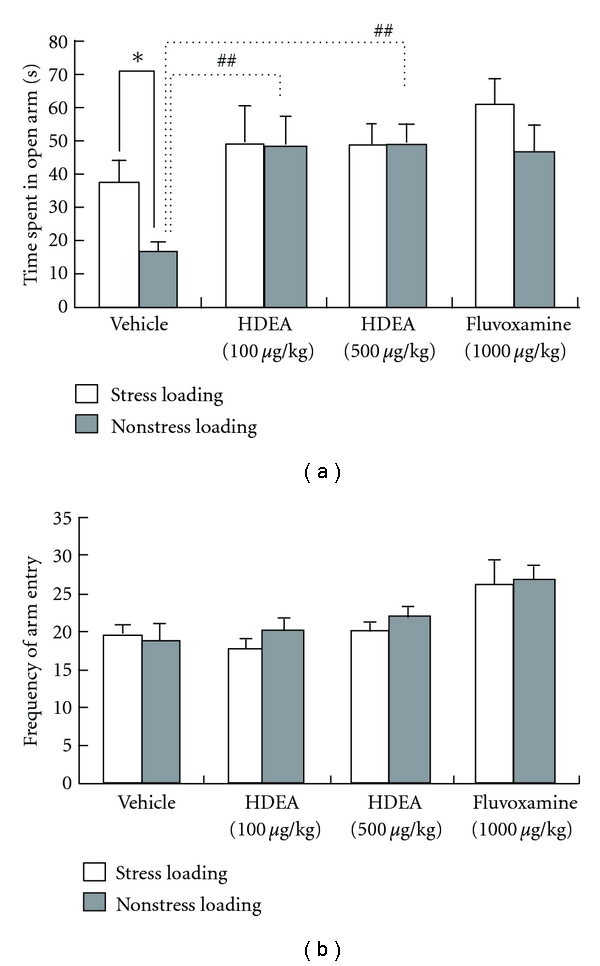
Effects of HDEA on the anxiety (a) and locomotor activity (b) in the elevated plus-maze test for mice. The mice were treated with vehicle, HDEA, or fluvoxamine as described in the legend of Figure 2 and then subjected to the elevated plus-maze test at 24 hr after the end of the stress loading. Significance of differences from the values of mice without stress loading was determined by use of Student's *t*-test (*P < 0.05 as indicated by the bracket). Significant differences from the value of the vehicle-treated mice with stress loading were determined by performing one-way ANOVA with Tukey's test (^##^ *P* < 0.01 as indicated by the brackets).

At first mice were assessed for the number of times each animal entered each of the arms and the time spent in each arm during a 5-min test period in the elevated plus-maze test before stress loading, and the outliers were excluded to minimize the variability of data among the subsequently treated groups. The remaining mice (*n* = 8/group) were administered vehicle, HDEA (100 *μ*g/kg), HDEA (500 *μ*g/kg), or fluvoxamine (1000 *μ*g/kg) once a day for 21 days during the stress loading. They were then given 5 min to explore the plus-shaped maze. The time spent in the open arms by the stress-loaded animals was markedly shorter than that for the nonstress-loaded ones in the vehicle-treated group, indicating that the stress induced an anxiety state (Figure 3(a)). However, no such significant difference was found in the low- and high-dose HDEA- and fluvoxamine-treated groups. Furthermore, the stress-loaded animals in either HDEA-treatment group spent significantly more time in the open arms compared with those in the vehicle group (Figure 3(a)). The finding that the frequency of entry into all arms was constant irrespective of stress loading in all experimental groups demonstrated that the locomotor activity was not influenced by the stress loading (Figure 3(b)). These observations suggest that HDEA was as effective as fluvoxamine to protect against the anxiety. 

**Figure 4:**
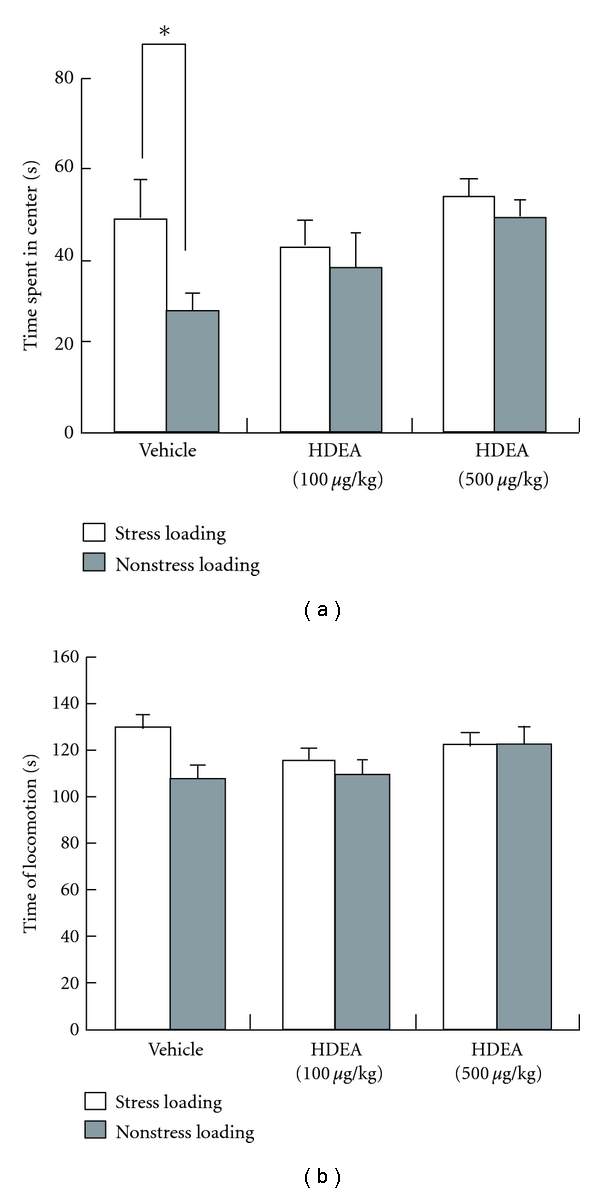
Effects of HDEA on the anxiety (a) and locomotor activity (b) in the open-field test for mice. The mice were treated with vehicle or HDEA as described in the legend of Figure 2 and then examined by the open-field test at 24 hr after the end of the stress. Significance of differences from the values of mice without stress loading was determined by using Student's *t*-test (*P < 0.05 as indicated by the bracket).

Another approach to evaluate the anxiety state is the open-field test. Initially, outliers in the open-field test were excluded before stress loading to minimize the variability of data among the subsequently treated groups. The retained mice (*n* = 8/group) were administered vehicle or 100 or 500 *μ*g/kg of HDEA once a day for 21 days during the stress loading. They were then given 5 min to explore an open field. The time spent in the central area by the stress-loaded animals was significantly shorter than that by the nonstress-loaded ones in the vehicle-treated group, indicating that the stress strengthened the anxiety state and resulted in a significant decrease in the time spent in the central area (Figure 4(a)). However, no significant difference was noted between stress-loaded and nonstress-loaded animals when the low- or high-dose HDEA was administered (Figure 4(a)). Locomotion time was unchanged irrespective of stress loading in all experimental groups (Figure 4(b)), demonstrating that the reduction in time spent in the central area was not related to locomotor activity. These observations suggest that HDEA was effective to protect against the anxiety also when evaluated by the open-field test.

### 3.3. Effect of RJ on the Stress-Induced Depression

**Figure 5:**
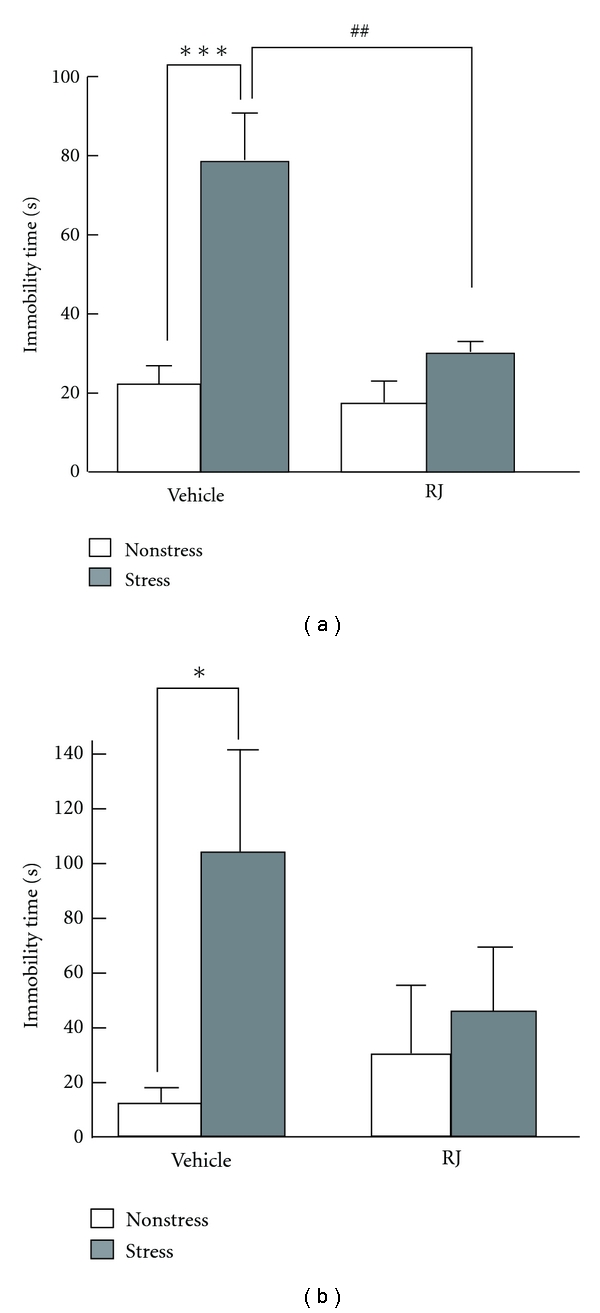
Effects of RJ on depression in mice. The tail-suspension test was used for evaluation of depression in mice injected with RJ intraperitoneally (a) or orally (b) once a day for 3 weeks with simultaneous exposure to the stress. Immobility values are expressed as the mean + SE (*n* = 3–5). Significance of differences from the values of the vehicle-treated mice without stress loading was determined by performing Student's *t*-test (*P < 0.05, ***P < 0.001 as indicated by the brackets). Significance of differences from the values of the vehicle-treated mice with stress exposure was determined by use of two-way ANOVA followed by the Bonferroni test (^##^ *P* < 0.01, as indicated by the brackets).

HDEA is uniquely and abundantly present in RJ [23]. Therefore, we examined the influence of RJ itself on the depression. The experiment was conducted in the same manner as that with HDEA or fluvoxamine (Figure 2). A dose of 250 mg/kg of RJ given by the intraperitoneal route significantly lowered the immobility time, which had been increased by stress in the vehicle group (Figure 5); however, much smaller doses of RJ did not have any significant effect (data not shown). RJ is a very popular health food and is generally taken orally. Therefore, finally we evaluated the effectiveness of orally administered RJ for protection against depression. An enormous large amount of RJ was needed for this purpose. The significant difference in immobility time between stressed and nonstressed vehicle-treated groups disappeared when RJ was taken orally at a dose of 2500 mg/kg, although no significant difference between the stressed RJ-treated and vehicle-treated groups was observed. These results suggest that orally administered RJ was less effective or much weaker than that given by the intraperitoneal route.

## 4. Discussion

Several results suggest that BDNF may play a protective role in the pathophysiology of depression [24]. Heterozygous BDNF knockout mice show behavioral abnormalities consistent with serotonergic dysfunction. Additional evidence connecting BDNF and depression comes from studies showing that infusion of recombinant BDNF into the mouse midbrain [25] or hippocampus [24] produces an antidepressant effect in both learned helplessness and forced swim models of depression. Furthermore, stress, a trigger for depression, lowers hippocampal transcription of BDNF in mice [26]. In contrast, numerous antidepressants, including selective serotonin reuptake inhibitors, electroconvulsive therapy, lithium, and monoamine oxidase inhibitors [26, 27], all increase BDNF transcription. This transcriptional increase occurs after a delay similar to that seen in the onset of clinical effects of antidepressants [26]. 

BDNF binds to a specific Trk receptor tyrosine kinase [28], which binding triggers signal transduction cascades including the pathways of mitogen-activated protein kinases (MAPK)/extracellular signal-regulated kinases (ERK)1/2 [29]. Activated ERK1/2 then passes into the nucleus to activate transcription factors such as cAMP response element binding protein (CREB), leading to regulation of expression of various genes involved in neuronal differentiation, learning, and memory [30, 31]. Intracellular signaling through ERK1/2 is important for the regulation of various cellular functions in the central nervous system. Analysis of gene-disrupted animals has clarified that ERK1/2 activity may govern neurogenesis to ensure proper brain development [32]. Therefore, activation of ERK1/2 is a key event to regulate neurogenesis. 

 Earlier we found that HDEA increases the generation of neurons and decreases that of glial cells from cultured NSCs [10]. This reciprocal response between neuronal and glial populations suggests that HDEA affects a neuronal lineage of NSCs having the ability to generate both neuronal and glial cells. We previously observed that BDNF similarly affects cultured NSCs [11]. Thus, HDEA supposedly commits neural progenitors to the fate of neuronal lineage similarly as BDNF. 

Previously we found that medium-chain fatty acids with 8–12 carbons and their esters facilitate the activation (phosphorylation) of ERK 1/2 of cultured embryonic cortical/hippocampal neurons [33]. In particular, trans-2-decenoic acid ethyl ester (DAEE) has the most potent activity. In that study we found that (1) DAEE stimulates phosphorylation of ERK1/2 via MEK activation; (2) DAEE activates CREB predominantly through ERK1/2 activation, not through other pathways such as cAMP/protein kinase A; (3) DAEE increases the expression of mRNAs of BDNF and neurotrophin-3 and the protein content of synapse-specific proteins such as synaptophysin, synapsin-1, and syntaxin [33]. From these observations, HDEA was considered to activate ERK1/2 followed by CREB phosphorylation, which might be a common target of BDNF and antidepressants to improve symptoms [34].

Dysfunctions of glucocorticoid receptors (GRs) have been implicated in the pathogenesis of stress-related depression [35, 36]. Antidepressants increase hippocampal neurogenesis, which action is followed by decreased depression and anxiety, via a GR-dependent mechanism involving GR phosphorylation and activation of a specific set of genes including BDNF gene [37]. Antidepressant-induced BDNF protein functions importantly to increase neurogenesis to ameliorate the symptoms of depression. However, Moutsatsou et al. [38] found that HDEA did not alter the glucocorticoid response element-mediated transcriptional activity, suggesting that GR-dependent mechanisms are not involved in the action mechanisms of HDEA. Therefore, the action mechanisms of HDEA are different from those of antidepressants currently used, because the antidepressant drugs are thought to activate the ERK1/2/CREB signaling pathway via antidepressant-induced BDNF. 

These results demonstrate that HDEA and RJ, a natural source of it, are effective in ameliorating the stress-inducible symptoms of depression and anxiety and suggest that they may become a promising tool as a new antidepressant.

## References

[1] Fujii A, Kobayashi S, Kuboyama N (1990). Augmentation of wound healing by royal jelly (RJ) in streptozotocin-diabetic rats. *Japanese Journal of Pharmacology*.

[2] Šver L, Oršolić N, Tadić Z, Njari B, Valpotić I, Bašić I (1996). A royal jelly as a new potential immunomodulator in rats and mice. *Comparative Immunology, Microbiology and Infectious Diseases*.

[3] Oka H, Emori Y, Kobayashi N, Hayashi Y, Nomoto K (2001). Suppression of allergic reactions by royal jelly in association with the restoration of macrophage function and the improvement of Th1/Th2 cell responses. *International Immunopharmacology*.

[4] Taniguchi Y, Kohno K, Inoue SI (2003). Oral administration of royal jelly inhibits the development of atopic dermatitis-like skin lesions in NC/Nga mice. *International Immunopharmacology*.

[5] Hattori N, Nomoto H, Mishima S (2006). Identification of AMP N_1_-oxide in royal jelly as a component neurotrophic toward cultured rat pheochromocytoma PC12 cells. *Bioscience, Biotechnology and Biochemistry*.

[6] Genç M, Aslan A (1999). Determination of trans-10-hydroxy-2-decenoic acid content in pure royal jelly and royal jelly products by column liquid chromatography. *Journal of Chromatography A*.

[7] Townsend GF, Brown WH, Felauer EE, Hazlett B (1961). Studies on the in vitro antitumor activity of fatty acids. IV. The esters of acids closely related to 10-hydroxy-2-decenoic acids from royal jelly against transplantable mouse leukemia. *Canadian Journal of Biochemistry and Physiology*.

[8] Koya-Miyata S, Okamoto I, Ushio S, Iwaki K, Ikeda M, Kurimoto M (2004). Identification of a collagen production-promoting factor from an extract of royal jelly and its possible mechanism. *Bioscience, Biotechnology and Biochemistry*.

[9] Blum MS, Novak AF, Taber S (1959). 10-hydroxy-delta 2-decenoic acid, an antibiotic found in royal jelly. *Science*.

[10] Hattori N, Nomoto H, Fukumitsu H, Mishima S, Furukawa S (2007). Royal jelly and its unique fatty acid, 10-hydroxy-trans-2-decenoic acid, promote neurogenesis by neural stem/progenitor cells in vitro. *Biomedical Research*.

[11] Ito H, Nakajima A, Nomoto H, Furukawa S (2003). Neurotrophins facilitate neuronal differentiation of cultured neural stem cells via induction of mRNA expression of basic helix-loop-helix transcription factors Mash1 and Math1. *Journal of Neuroscience Research*.

[12] Gould E, Reeves AJ, Graziano MSA, Gross CG (1999). Neurogenesis in the neocortex of adult primates. *Science*.

[13] Sheline YI (2000). 3D MRI studies of neuroanatomic changes in unipolar major depression: the role of stress and medical comorbidity. *Biological Psychiatry*.

[14] Malberg JE, Eisch AJ, Nestler EJ, Duman RS (2000). Chronic antidepressant treatment increases neurogenesis in adult rat hippocampus. *Journal of Neuroscience*.

[15] Duman RS, Heninger GR, Nestler EJ (1997). A molecular and cellular theory of depression. *Archives of General Psychiatry*.

[16] Ito N, Nagai T, Yabe T, Nunome S, Hanawa T, Yamada H (2006). Antidepressant-like activity of a Kampo (Japanese herbal) medicine, Koso-san (Xiang-Su-San), and its mode of action via the hypothalamic-pituitary-adrenal axis. *Phytomedicine*.

[17] Detke MJ, Johnson J, Lucki I (1997). Acute and chronic antidepressant drug treatment in the rat forced swimming test model of depression. *Experimental and Clinical Psychopharmacology*.

[18] Porsolt RD, Bertin A, Jalfre M (1977). Behavioral despair in mice: a primary screening test for antidepressants. *Archives Internationales de Pharmacodynamie et de Thérapie*.

[19] Willner P, Towell A, Sampson D, Sophokleous S, Muscat R (1987). Reduction of sucrose preference by chronic unpredictable mild stress, and its restoration by a tricyclic antidepressant. *Psychopharmacology*.

[20] Vale AL, Green S, Montgomery AMJ, Shafi S (1998). The nitric oxide synthesis inhibitor L-NAME produces anxiogenic-like effects in the rat elevated plus-maze test, but not in the social interaction test. *Journal of Psychopharmacology*.

[21] Bhattacharya SK, Satyan KS, Chakrabarti A (1997). Anxiogenic action of caffeine: an experimental study in rats. *Journal of Psychopharmacology*.

[22] Gadotti VM, Bonfield SP, Zamponi GW Depressive-like behavior of mice lacking cellular prion protein.

[23] Takenaka T (1982). Chemical composition of royal jelly. *Honeybee Science*.

[24] Duman RS (2002). Synaptic plasticity and mood disorders. *Molecular Psychiatry*.

[25] Siuciak JA, Lewis DR, Wiegand SJ, Lindsay RM (1996). Antidepressant-like effect of brain-derived neurotrophic factor (BDNF). *Pharmacology Biochemistry and Behavior*.

[26] Nibuya M, Morinobu S, Duman RS (1995). Regulation of BDNF and trkB mRNA in rat brain by chronic electroconvulsive seizure and antidepressant drug treatments. *Journal of Neuroscience*.

[27] Russo-Neustadt A, Beard RC, Cotman CW (1999). Exercise, antidepressant medications, and enhanced brain derived neurotrophic factor expression. *Neuropsychopharmacology*.

[28] Barbacid M (1995). Structural and functional properties of the TRK family of neurotrophin receptors. *Annals of the New York Academy of Sciences*.

[29] Kaplan DR, Miller FD (2000). Neurotrophin signal transduction in the nervous system. *Current Opinion in Neurobiology*.

[30] Huang EJ, Reichardt LF (2001). Neurotrophins: roles in neuronal development and function. *Annual Review of Neuroscience*.

[31] Lu Q, Hutchins AE, Doyle CM, Lundblad JR, Kwok RPS (2003). Acetylation of cAMP-responsive element-binding protein (CREB) by CREB-binding protein enhances CREB-dependent transcription. *Journal of Biological Chemistry*.

[32] Satoh Y, Kobayashi Y, Takeuchi A, Pagès G, Pouysségur J, Kazama T (2011). Deletion of ERK1 and ERK2 in the CNS causes cortical abnormalities and neonatal lethality: Erk1 deficiency enhances the impairment of neurogenesis in Erk2-deficient mice. *Journal of Neuroscience*.

[33] Makino A, Iinuma M, Fukumitsu H, Soumiya H, Furukawa Y, Furukawa S (2010). 2-decenoic acid ethyl ester possesses neurotrophin-like activities to facilitate intracellular signals and increase synapse-specific proteins in neurons cultured from embryonic rat brain. *Biomedical Research*.

[34] Hyman SE, Nestler EJ (1996). Initiation and adaptation: a paradigm for understanding psychotropic drug action. *American Journal of Psychiatry*.

[35] Holsboer F (2000). The corticosteroid receptor hypothesis of depression. *Neuropsychopharmacology*.

[36] Nestler EJ, Barrot M, DiLeone RJ, Eisch AJ, Gold SJ, Monteggia LM (2002). Neurobiology of depression. *Neuron*.

[37] Anacker C, Zunszain PA, Cattaneo A (2011). Antidepressants increase human hippocampal neurogenesis by activating the glucocorticoid receptor. *Molecular Psychiatry*.

[38] Moutsatsou P, Papoutsi Z, Kassi E (2010). Fatty acids derived from royal jelly are modulators of estrogen receptor functions. *PLoS One*.

